# Fumonisin B_1_ (FB_1_) Induces Lamellar Separation and Alters Sphingolipid Metabolism of *In Vitro* Cultured Hoof Explants

**DOI:** 10.3390/toxins8040089

**Published:** 2016-03-24

**Authors:** Nicole Reisinger, Ilse Dohnal, Veronika Nagl, Simone Schaumberger, Gerd Schatzmayr, Elisabeth Mayer

**Affiliations:** BIOMIN Research Center, Tulln 3430, Austria; ilse.dohnal@biomin.net (I.D.); veronika.nagl@biomin.net (V.N.); simone.schaumberger@biomin.net (S.S.); gerd.schatzmayr@biomin.net (G.S.); e.mayer@biomin.net (E.M.)

**Keywords:** horses, fumonisin, hoof explants, laminitis, Sa/So ratio

## Abstract

One of the most important hoof diseases is laminitis. Yet, the pathology of laminitis is not fully understood. Different bacterial toxins, e.g. endotoxins or exotoxins, seem to play an important role. Additionally, ingestion of mycotoxins, toxic secondary metabolites of fungi, might contribute to the onset of laminitis. In this respect, fumonsins are of special interest since horses are regarded as species most susceptible to this group of mycotoxins. The aim of our study was to investigate the influence of fumonisin B_1_ (FB_1_) on primary isolated epidermal and dermal hoof cells, as well as on the lamellar tissue integrity and sphingolipid metabolism of hoof explants *in vitro*. There was no effect of FB_1_ at any concentration on dermal or epidermal cells. However, FB_1_ significantly reduced the separation force of explants after 24 h of incubation. The Sa/So ratio was significantly increased in supernatants of explants incubated with FB_1_ (2.5–10 µg/mL) after 24 h. Observed effects on Sa/So ratio were linked to significantly increased sphinganine concentrations. Our study showed that FB_1_ impairs the sphingolipid metabolism of explants and reduces lamellar integrity at non-cytotoxic concentrations. FB_1_ might, therefore, affect hoof health. Further *in vitro* and *in vivo* studies are necessary to elucidate the effects of FB_1_ on the equine hoof in more detail.

## 1. Introduction

Hoof related diseases are a common cause of lameness in horses [1]. One of those diseases is laminitis, which causes severe damage of the lamellar tissue and detachment of the pedal bone. The pathology of laminitis is yet not fully understood and bacterial toxins, such as endotoxins [2,3] or exotoxins [4,5], are discussed to play an important role. In addition, ingestion of mycotoxins, toxic secondary metabolites of fungi, might contribute to the onset of laminitis. For example, ergovaline, a mycotoxin produced by endophyte-infected fescues, negatively affects hoof health *in vitro* [6] and *in vivo* [7,8] by vasoconstriction. Unfortunately, no information on the effects of other mycotoxins on the equine hoof is available.

In this respect, fumonisins might be of special interest since horses are regarded as a species most susceptible to these mycotoxins. Fumonisins are a group of mycotoxins mainly produced by *Fusarium* spp., such as *F. verticillioides* and *F.* *proliferatum*. Among different analogues identified so far, fumonisin B1 (FB_1_) is the most relevant, considering prevalence and toxicity [9]. FB_1_ is a frequent contaminant of corn, wheat, barley, oats, and hay and can be detected in more than 90% of commercial horse feeds [10]. Hence, horses are at high risk to ingest FB_1_ and to suffer from its effects. Due to its structural similarity to the sphingoid bases sphinganine (Sa) and sphingosine (So), FB_1_ inhibits the *de novo* ceramide synthesis and, thereby, alters the sphingolipid metabolism. As a consequence, FB_1_ induces a broad range of adverse health effects, including liver and kidney toxicity. In horses, FB_1_ causes a specific disease called equine leukoencephalomalacia (ELEM) [11], which is characterized by neurological symptoms (e.g., circling, head pressing, ataxia), hepatotoxicity, and leads to irreversible damage of the brain [12,13,14]. Furthermore, an increase of the sphinganine to sphingosine (Sa/So) ratio was observed in serum. As these changes occurred prior to alterations of liver enzymes, the Sa/So ratio might be an early biomarker for FB_1_ exposure in horses [15,16], indicating impaired ceramide biosynthesis. As ceramides are important parts of the equine hoof tissue, fumonisins might also affect equine hoof health. One report [17] already described a significant difference in total ceramide content of claws of healthy cows and cows with subclinical laminitis.

Still, effects of FB_1_ on equine hoof health have not been investigated so far. Thus, the aim of our study was to assess the cytotoxic effects of FB_1_ on primary isolated epidermal and dermal hoof cells. Furthermore, the impact of increasing FB_1_ concentrations on the lamellar tissue integrity and sphingolipid metabolism was evaluated *in vitro*. This study will provide first results on the effects of FB_1_ on primary hoof cells and hoof tissue *in vitro* and its potential role in hoof related diseases.

## 2. Results

### 2.1. Cytotoxicity of FB_1_ on Primary Hoof Cells

**Figure 1:**
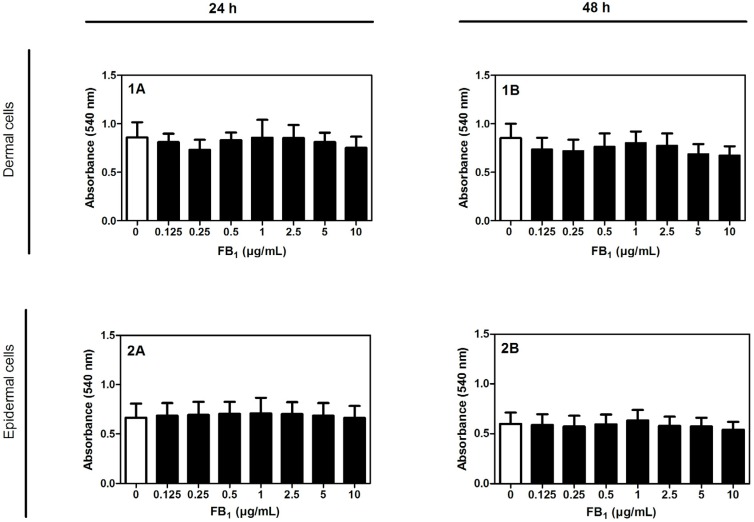
Absorbance values (540 nm) measured with the neutral red assay to evaluate the viability of dermal hoof cells (1) and epidermal hoof cells (2) incubated with different concentrations of FB_1_ (0–10 µg/mL) for (**A**) 24 h and (**B**) 48 h (*n* = 4). Error bars present standard deviation.

To ensure that used FB_1_ concentrations (0.125–10 µg/mL) do not have any cytotoxic effects, the neutral red viability assay was performed with primary hoof cells after 24 and 48 h of incubation. FB_1_ (0.125–10 µg/mL) had no cytotoxic effect on dermal or on epidermal cells after 24 h and 48 h, respectively (Figure 1).

### 2.2. Viability and Separation Force of Control Hoof Explants

**Figure 2:**
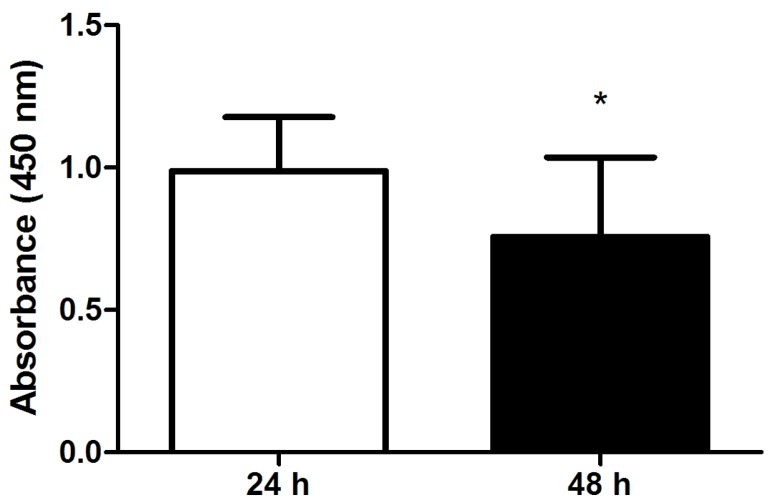
Absorbance values (450 nm) measured with the WST-1 assay to evaluate the viability of explants incubated with culture medium (control, no FB_1_ addition) for 24 h (*n* = 12) and 48 h (*n* = 9). Error bars present standard deviation. * *p* < 0.05.

To assess a potential effect of incubation time on investigated parameters, viability (WST-1 assay) and separation force of explants incubated in medium for 24 h (*n* = 12 explants) or 48 h (*n* = 9 explants) were compared. Hoof explants incubated for 48 h showed a significantly decreased viability compared to explants incubated for 24 h (Figure 2).

There was no significant decrease of separation force of explants incubated for 48 h compared to explants incubated for 24 h (*p* = 0.1452).

### 2.3. Effect of FB_1_ on Separation Force

The effects of different FB_1_ concentrations (0–10 µg/mL) on hoof tissue integrity was tested. To this end, a force transducer was used to measure the force needed to separate the explants after incubation with FB_1_ for 24 h (*n* = 12 explants) and 48 h (*n* = 9 explants).

There was a significant influence of incubation time (*p* = 0.0010) and FB_1_ (*p* = 0.006) on separation force. No interaction of both factors was observed (*p* = 0.905).

Compared to the controls, a significantly decreased separation force was observed in explants incubated with FB_1_ for 24 h (Table 1). As an exception, incubation with 0.5 µg/mL FB_1_ did not lead to a reduced separation force.

After 48 h, only explants incubated with 1 µg/mL FB_1_ showed a significantly decreased separation force (Table S1). Incubation of explants with 10 µg/mL lipopolysaccharides (LPS; positive control) for 24 h led to a significant decrease of separation force (Figure S1).

### 2.4. Effects of FB_1_ Sphingolipid Metabolism

A LC-MS/MS method was used to determine the Sa and So concentrations in explant supernatants (*n* = 8) incubated for 24 h with FB1 (0–10 µg/mL).

**Figure 3:**
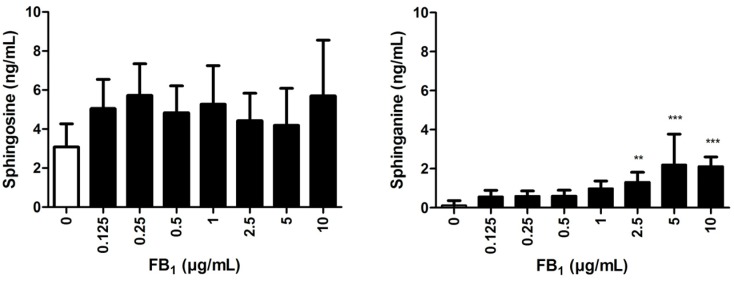
Sphingosine and sphinganine supernatant concentration of explants incubated with FB_1_ (0–10 µg/mL) for 24 h (*n* = 8). Error bars present standard deviation. ** *p* < 0.01; *** *p* < 0.001 compared to explants incubated without FB_1_.

A significant increase of the Sa concentration could be observed in supernatants of explants incubated with 2.5–10 µg/mL FB_1_ (Figure 3). There was no significant influence of any FB_1_ concentration on the So concentration.

**Figure 4:**
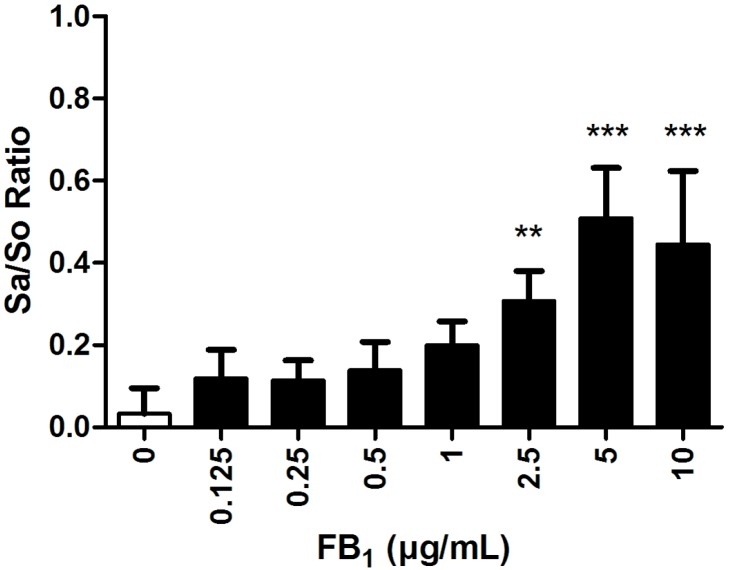
Sphinganine to sphingosine (Sa/So) ratio of explants incubated with FB_1_ (0–10 µg/mL) for 24 h (*n* = 8). Error bars present standard deviation. ** *p* < 0.01; *** *p* < 0.001 compared to explants incubated without FB_1_.

The Sa/So ratio was significantly increased in supernatants of explants incubated with 2.5–10 µg/mL FB_1_ (Figure 4). A concentration-dependent effect could be observed from 0.5 µg/mL and higher, except for explants incubated with 10 µg/mL.

## 3. Discussion

The effects of mycotoxins in horses are poorly investigated compared to other species, e.g., pigs, broilers, or cows. Fumonisins have gathered certain interest in equine science due the detrimental effects of FB_1_ in horses. Still, only some case reports and a few scientific studies on the impact of FB_1_ on equine health are available. A recent study in 2015 reported an outbreak of fumonisin toxicosis in horses in Serbia [18]. Feed components such as maize and maize bran contained 8400 µg/kg and 7730 µg/kg fumonisins, respectively. Of 21 horses affected, 15 died and showed brain lesions typical for ELEM. There are several other publications, which reported cases of ELEM in horses in Europe, United States, and South Africa [11,19,20]. It has to be mentioned that concentrations of 1000 µg/kg can lead to symptoms of ELEM or death in horses [19]. Mycotoxin surveys demonstrate frequent occurrence of fumonisins in feedstuffs. Streit *et al.* [21] observed that 53% of feed and feed raw material were positive for fumonisins with a maximum concentration of 77,500 µg/kg. Another study by Liesner *et al.* [10] found 93% of commercial available horse feed preparations (*n* = 62) positive for fumonisins with a maximum concentration of 2200 µg/kg. Furthermore, effects of fumonisins might be potentiated by the co-occurrence of other mycotoxins in feed. Intriguingly, individual feed samples were reported to contain up to 69 fungal metabolites [22]. For the majority of these metabolites, no toxicological data in horses are available. In addition, the impact of long-term exposure to subclinical concentrations of FB_1_ (potentially affecting performance, fertility, or general health status) and the effects of FB_1_ on the equine hoof are yet unknown.

Hoof diseases are one of the most common reasons for lameness in horses. Laminitis, which is the most frequent hoof disease, affects up to 34% of horses [23]. In the United States alone, the monetary loss per year is estimated to be around $13 million. These losses include evaluation, treatment, and euthanization of horses after a diagnosis of laminitis [24]. One of the greatest challenges in preventing and treating laminitis is the multifactorial etiology. There is still a lack of knowledge about the main trigger factors and pathology of this disease. The study, therefore, focused on the effects of FB_1_ on hoof health.

First, the cytotoxic potential on dermal and epidermal hoof cells of FB_1_, the most relevant fumonisin derivative [25], was evaluated. Cells were isolated from hooves as there is no suitable equine cell line commercially available. Our results showed that there is no effect of FB_1_ concentrations up to 10 µg/mL (~14 µM) on dermal or epidermal cells for 24 and 48 h. Unfortunately, there are no reports available on the cytotoxic potential of FB_1_ in other equine cell lines. In general, the effects of FB_1_ seem to be dependent on factors such as concentrations used, cell types/line, and whether FB_1_ is added to proliferating or confluent cells. For example, FB_1_ did not evoke cytotoxicity in rat primary hepatocytes (50 µM, [26]), porcine primary endothelial cells (10–50 µM [27]), human fibroblasts (10–100 µM, [28]), or human astrocytes (10–100 µM, [29]). Notably, Kouadio *et al.* [30] determined no cytotoxic effects of FB_1_ in human intestinal cells with the neutral red assay, at concentrations up to 150 µM FB_1_, but measured an effect with the MTT assay at 21 µM FB_1_. These differences might be related to the assays focusing on different cell targets, thereby highlighting the relevance of the employed test system. As we could not observe an effect of FB_1_ on the viability of hoof cells with the neutral red assay as well, it will be interesting to investigate the cytotoxicity with assays focusing different cell targets. In addition, FB_1_ might induce apoptosis in hoof cells, which cannot be measured with the applied assay. Dietary exposure of rats to high FB_1_ concentrations (99–448,000 µg/kg) induced apoptosis in the kidney and liver of rats [31]. Furthermore, FB_1_ induced apoptosis in human neonatal keratinocytes and fibroblasts, in combination with decreased cell viability [32,33]. Hence, it will be important to assess the effect of FB_1_ on the number of apoptotic hoof cells.

As a next step, the influence of FB_1_ on the separation force of hoof explants was tested. The explant model offers the advantage to test a lot of different concentrations at once. However, one limitation of explant models is the limited lifespan of hoof tissue *in vitro*. Often, viability is not assessed in studies using explants, although this is rather important to ensure that decreased viability has no effect on obtained results. Our data showed that viability of explant was significantly decreased after 48 h, which confirms previous findings of our group [34]. Hence, we can conclude that explants should only be cultivated for 24 h as the lifespan of explants *in vitro* seems to be limited. Decreased viability will potentially affect other parameters as separation force and, therefore, experiments with extended incubation time should be evaluated with care.

When adding FB_1_ for 24 h to the explants, we measured a significant decrease of the separation force. LPS was used as positive control to validate the hoof explant model. Other studies have already shown that LPS is able to reduce separation force of explants [34,35]. As expected, also in our study incubation with 10 µg/mL LPS led to a significant reduction of separation force. At the moment, we cannot conclude on the definite cause that led to the FB_1_-mediated lamellar separation. There are only studies available showing a decrease in the separation force when explants were incubated with exotoxins [4,5] or endotoxins [34,35]. Exotoxins led to an increased matrix metalloproteinase (MMP) −2 and −9 activity, which leads to the separation of explants [4,36]. However, other MMPs in the lamellar tissue such as MMP-13 might also play an important role [37]. Beside possible activation of different enzymes like MMPs, FB_1_ might initiate inflammation processes, which cause destruction of the lamellar tissue. A study by Mammodi *et al.* [38], for example, showed that FB_1_ can induce inflammatory cytokine response in a gastric epithelial and a colon adenocarcinoma cell line.

In addition to lamellar tissue integrity, we evaluated the influence of FB_1_ on sphingolipid metabolism in explants. The Sa/So ratio was determined in supernatants of explants incubated with FB_1_ for 24 h only, due to the decreased viability of the control explants after 48 h incubation. We measured a significantly increased Sa/So ratio in supernatants of explants treated with FB_1_ (0.5–10 µg/mL). This effect was also concentration dependent except for explants treated with 10 µg/mL FB_1_ after 24 h. The observed effects on the Sa/So ratio in our study were linked to an increase of sphinganine concentration in explant supernatants. In many species, the Sa/So ratio has been described as sensitive biomarker for FB_1_ exposure [39,40]. FB_1_ is known to inhibit ceramide synthase, which, as a consequence, leads to intracellular accumulation of Sa, and also (to a minor extent) So. Accordingly, an increase of the serum Sa/So ratio was observed in horses exposed to 15,000–44,000 µg/kg FB_1_. This parameter might already be increased before clinical signs or alterations in serum levels of liver enzymes can be detected [15].

In our study, the disruption of the ceramide synthesis could be an explanation for the decreased lamellar tissue integrity we observed after FB_1_ exposure. Ceramides are important parts of the equine hoof tissue and can be found, e.g., in the intracellular spaces of keratinocytes [41]. The keratinized layer of the hoof tissue has an important role as effective barrier against environmental impacts and prevents water loss. There are no studies available which evaluated the effects of ceramide concentration on the pathology of hoof related diseases in horses. However, a study by Higuche *et al.* [17] described a significant correlation between ceramide concentration and hardness of the claws in healthy cows and cows with subclinical laminitis. Furthermore, a significant decrease in the hardness of the claw of cows with subclinical laminitis could also be observed. As keratinocytes synthesize ceramides, the decrease during subclinical laminitis might be caused by changes of the keratinocyte function due to inflammatory processes.

In general, *in vitro* systems are often less sensitive compared to *in vivo* studies as they present an isolated system (highly-controlled conditions) in the absence of other trigger factors. This is of special importance when it comes to the multifactorial etiology of laminitis.

FB1 concentrations used in our study were similar or even lower (0.17–13.85 µM) compared to those applied in other *in vitro* studies (5–100 µM). Even in *in vivo* studies, comparably high FB1 concentrations (exceeding the recommendation level for horses of 5,000 µg FB1/kg feed) are regularly used. Although such experiments might not always represent the conditions in the field they are, nevertheless, crucial to understand the mode of action of FB1.

It is difficult to compare the FB1 concentrations used in our study to the situation *in vivo*, as there are only a few publications available about recovery from the blood or from specific tissue. For example, Fodor *et al.* [42] detected 17.4 µg/kg FB1 accumulated in the liver, when piglets were fed with 45,000 µg/kg FB_1_ for 10 days. Administration of 10,000 µg/kg FB_1_ *per os* in rats led to plasma concentrations of 0.180 µg/mL [43]. In vervet monkeys, also concentrations of up to 0.210 µg/mL FB_1_ in the plasma could be measured after gavage of 6420 µg/kg body weight FB_1_ [44]. A single administration of 5000 µg/kg FB_1_ *per os* in weaning piglets led to plasma concentrations of 0.300 µg/mL after 2 h [45]. About half of this concentration (0.125 µg/mL) FB_1_ already significantly decreased separation force in our study and an eight times higher concentration led to a significant increase of the Sa/So ratio.

FB_1_ induced disruption of the ceramide metabolism might, therefore, cause structural changes in the explants, subsequently leading to a decreased separation force. However, the mechanism which induces lamellar destruction by FB_1_ has to be further investigated.

## 4. Conclusions

Our study indicates that FB_1_ has no cytotoxic effect on equine epidermal and dermal cells but impairs the sphingolipid metabolism of explants and reduces lamellar integrity. FB_1_ is probably not capable of inducing laminitis itself but it might have an effect on the development or on the severity of laminitis. Although further *in vitro* and *in vivo* studies are necessary to elucidate the effects of FB_1_ on the equine hoof in more detail, the potential impact of fumonisins on equine health was, once again, highlighted.

## 5. Materials and Methods

### 5.1. Preparation of FB_1_ Stock Solution

Solid FB_1_ standard was purchased from Romer Labs (Tulln, Austria). The stock solution, containing 1168 µg/mL FB_1_, was prepared in distilled water, sterile filtrated (0.2 µm filter), and stored at 4 °C. FB_1_ stock solution was diluted with culture medium to yield a concentration of 0.125–10 µg/mL FB_1_ in the wells. Negative influence of solvent on explants was excluded.

### 5.2. Ethical Statement

Equine hooves were obtained from a local abattoir. No horse was killed for the purpose of tissue collection.

### 5.3. Animals

Forelimbs from five adult horses were obtained at a local abattoir. There was no information of age, gender, breed, or history of the horses available. Horses were killed by use of a penetrating captive bolt and subsequent exsanguination. Only forelimbs with healthy hooves were processed. The condition of hooves was assessed by macroscopic examination by a veterinarian and microscopic evaluation of lamella tissue. Isolated forelimbs were transported on ice to the laboratory. Transportation time to the laboratory did not exceed 120 min.

### 5.4. Preparation of Hoof Explants

Hooves were prepared and dissected as described in the literature by Reisinger *et al.* [34]. Immediately after preparation, explants were either used for isolation of primary hoof cells (cytotoxicity assays) or for direct incubation with different FB_1_ concentrations (lamellar separation tests).

### 5.5. Isolation, Cultivation, and Cytotoxicity Tests of Dermal and Epidermal Cells

Isolation of dermal and epidermal cells was performed as described by Reisinger *et al.* [35].

Cells were passaged and grown in T-75 flasks (Star Lab, Hamburg, Germany). Dermal cells were used until passage 18, while epidermal cells were only used until passage 10. Cells were seeded in 96 well plates (Eppendorf, Vienna, Austria) with a density of 4 × 10^4^ cells and 200 µL medium per well. After two days, cells were incubated with FB_1_ (0–10 µg/mL) in triplicate for 24 and 48 h. Four independent experiments were performed for each incubation time and cell type, respectively.

The neutral red assay was performed according to the instructions of the manufacturer (Aniara, Columbus, OH, USA) to determine the cytotoxicity of increasing FB_1_ concentrations on epidermal and dermal cells. This viability assay is based on the ability of viable cells to incorporate and bind neutral red within lysosomes.

### 5.6. Cultivation, Viability, and Lamellar Separation (Force Transducer) of Hoof Explants

Explants were cultured with 1 mL medium at 37 °C and 5% CO_2_ in 24 well plates (IWAKI, Willich, Germany). D-MEM (4.5 g/L glucose; Life technologies, Vienna, Austria) supplemented with 100 U/mL nystatin (Life technologies) and 0.1 mg/L gentamicin (Life technologies) was used as the culture medium. Explants were cultured with different FB_1_ concentrations (0–10 µg/mL) for 24 h (quadruplicate per horse) or 48 h (triplicate per horse). As positive control, explants were incubated with 10 µg/mL lipopolysaccharides from *Escherichia coli* O55:B5 (Sigma, Vienna, Austria) [34] for 24 h (quadruplicate per horse). Thereafter, all explants were examined via microscope before proceeding with testing. Explants showing bacterial or fungal contamination were excluded from the results.

Explants were tested for their viability with the water soluble tetrazolium (WST-1) as described by Reisinger *et al.* [34]. A comparison between control explants (0 µg/mL FB_1_) for 24 (*n* = 12) and 48 h (*n* = 9) was performed. The force required for explant separation was measured by a calibrated force transducer as described by Reisinger *et al.* [34].

### 5.7. Sphinganine (Sa) and Sphingosine (So) Analysis via LC-MS/MS

Supernatants of explants incubated with FB_1_ (0–10 µg/mL) for 24 h were collected under sterile conditions in reaction tubes (Eppendorf). Tubes were stored at −20 °C until further processing. Supernatants were thawed; proteins were precipitated by addition of the four-fold volume of methanol (Sigma), and removed by centrifugation at 20,000× *g* for 10 min at room temperature. Thereafter, supernatants were evaporated to dryness in a SpeedVac (Genevac, Ipswich, UK) and reconstituted in 80% (*v*/*w*) methanol to the original sample volume.

Sa and So were analyzed via LC–MS/MS as described by Grenier *et al.* [46] with some modifications. A Kinetex C18 column (2.6 µm, 100 Å 150 × 2.1 mm; Phenomenex, Torrance, CA, USA) was used for chromatographic separation on an Agilent 1290 series UHPLC system (Agilent, Waldbronn, Germany), with a flow rate of 0.25 mL/min and a linear gradient from 35% B to 100% B (eluent A: methanol/water/formic acid, 40/59.85/0.15 *v*/*v*/*v*; eluent B: methanol/formic acid 99.85/0.15). The injection volume was set to 2 µL. Tandem mass spectrometric detection was carried out on a 5500 Triple Quad system (AB Sciex, Framingham, MA, USA) after electrospray ionization in positive mode, using the following source parameters: curtain gas 40, collision gas 7, ion spray voltage 5500 V, source temperature 550 °C , GS1 50, GS2 50. The declustering potentials (DP), quantifier (quant) and qualifier (qual) transitions were DP 71 V, quant: *m/z* 300.3 to 282.3 (CE 15 eV), qual: *m/z* 300.3 to 252.2 (CE 23 eV) for So and DP 146 V, quant: *m/z* 302.3 to 284.3 (CE 19 eV), qual: *m/z* 302.3 to 60.1 (CE 21 eV) for Sa. Dwell time for all transitions was 50 ms. Analyst software version 1.6.2 (AB Sciex, 2013) was used for instrument control and data evaluation. Sa and So standards for external standard calibration functions were purchased form Avanti Polar Lipids, Inc. (Alabaster, AL, USA). The overall recovery was 90% ± 8% for Sa and 87% ± 8% for So, while the limit of quantitation was 0.3 µg/L for both analytes.

### 5.8. Statistical Analyses

Statistical evaluation was performed with IBM SPSS Statistics software (Version 19.0, IBM corp., New York, NY, USA, 2010). If data were normally distributed (Kolmogorov-Smirnov test), either *t*-test (unpaired, two-tailed) or ANOVA was performed with Dunnett’s test as a *post* *hoc* test. If data were not normally distributed, the Kruskal-Wallis test was used as a non-parametric test. Results were considered significant at *p* < 0.05.

## Figures and Tables

**Table 1:** Mean separation force (Newton (N)) of explants incubated with FB_1_ (0–10 µg/mL) for 24 h (*n* = 12).

24 h			
FB_1_ (µg/mL)	Mean (N)	SD	*p*-Value
0	21.0	8.8	-
0.125	13.4	7.1	0.007
0.25	13.1	9.1	0.016
0.5	12.6	4.1	0.084
1	11.5	6.1	0.000
2.5	12.7	5.6	0.003
5	12.9	6.4	0.004
10	14.3	7.7	0.005

## Supplementary Materials

The following are available online at www.mdpi.com/2072-6651/8/4/89/s1, Table S1: Mean separation force (N) of explants incubated with FB_1_ (0–10 µg/mL) for 48 h (*n* = 9), Figure S1: Comparison of the separation force of explants treated with 10 µg/mL LPS (positive control) to control explants (negative control).

## Abbreviations

The following abbreviations are used in this manuscript:

ELEM	Equine Leukoencephalomalacia
FB_1_	Fumonisin B_1_
LPS	Lipopolysaccharides
Sa	Sphinganine
So	Sphingosine
Sa/So ratio	Sphinganine-to-sphingosine ratio
WST	Water Soluble Tetrazolium
