# Supplementary material for: Fumonisin B1 (FB1) Induces Lamellar Separation and Alters Sphingolipid Metabolism of In Vitro Cultured Hoof Explants
Source: Toxins (Basel). 2016 Mar 24;8(4):89. doi: 10.3390/toxins8040089 (PMC4848616; doi:10.3390/toxins8040089)
Supplement: Supplementary file 1 [file toxins-08-00089-s001.pdf]

Supplementary Materials: Fumonisin B1 (FB1) Induces Lamellar Separation and Alters Sphingolipid Metabolism of *In Vitro* Cultured Hoof Explants

Nicole Reisinger *, Ilse Dohnal, Veronika Nagl, Simone Schaumberger, Gerd Schatzmayr and Elisabeth Mayer

Table S1. Mean separation force (N) of explants incubated with FB1 (0–10 µg/mL) for 48 h (n = 9).

48 h			
**FB1 (µg/mL)**	**Mean (N)**	**SD**	***p*-Value**
0	14.4	7.5	-
0.125	n.a.	n.a.	n.a.
0.25	n.a.	n.a.	n.a.
0.5	10.1	4.3	0.415
1	7.8	6.6	0.045
2.5	7.7	3.7	0.083
5	8.8	5.9	0.193
10	11.8	6.6	0.834

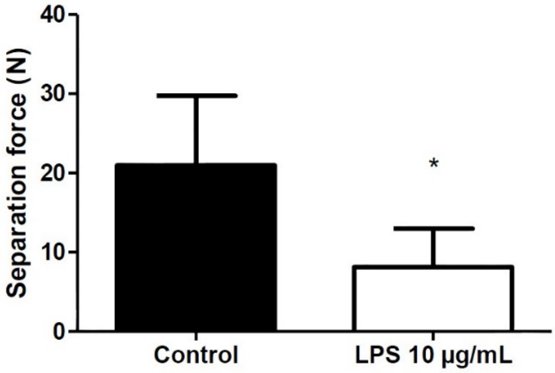

Figure S1. Comparison of the separation force of explants treated with 10 µg/mL LPS (positive control) to control explants (negative control). * $p < 0.05$.
